# Is befriending a valuable intervention in schizophrenia? A scoping review

**DOI:** 10.3389/fpsyt.2023.1189772

**Published:** 2023-06-02

**Authors:** Adriana Farcas, Mackenzie Campbell, Charmaine Wong, Felicia Iftene

**Affiliations:** Providence Care Hospital, Queen’s University, Kingston, ON, Canada

**Keywords:** befriending, schizophrenia, CBT, psychosocial, psychiatry

## Abstract

**Background:**

Schizophrenia is a severe, chronic mental disorder that involves disruptions in cognitive processes, emotional responsiveness, and social interactions. Psychotherapeutic and social integration practices have increasingly been added to the pharmacological treatment in an effort to improve the level of functioning and the quality of life of individuals affected by this condition. Befriending, defined as a one-on-one companionship provided by a volunteer who aims to act as an emotionally supportive liaison, is hypothesized to be an effective such intervention, offering support for building and maintaining social relationships in the community. Despite its increase in popularity and acceptance, befriending remains poorly understood and under-researched.

**Methods:**

We performed a systematic search for studies targeting befriending either as an intervention or a controlled condition in studies on schizophrenia. Searches were performed in four databases: APA PsycInfo, Pubmed, Medline and EBSCO. The keywords “schizophrenia,” AND “befriending,” were searched for on all databases.

**Results:**

The search yielded 93 titles and abstracts, of which 18 met the criteria for inclusion. The studies included in this review have all incorporated befriending as an intervention or a controlled condition, as per our search criteria, and aimed at depicting the value and feasibility of this intervention to address social and clinical deficits in individuals with schizophrenia.

**Conclusion:**

The studies selected for this scoping review revealed inconsistent findings regarding the effect of befriending on overall symptoms and the subjective reporting of quality of life in individuals with schizophrenia. This inconsistency may be attributed to differences between the studies and their specific limitations.

## Introduction

Schizophrenia is a severe, chronic mental disorder that involves disruptions in cognitive processes, emotional responsiveness, and social interactions. Individuals with schizophrenia tend to exhibit poor social functioning and are characteristically more socially isolated when compared to other groups of people in the general population (1). Psychotherapeutic and social integration practices have increasingly been included in therapeutic guidelines in addition to the pharmacological treatment, to improve quality of life. A recent systematic review of efficacy meta-analyses on psychosocial and behavioral interventions in schizophrenia (2) highlights the most cited ones in the literature: CBT and cognitive based interventions, social skills training, acceptance, and mindfulness based approaches, family interventions, exercise therapy, music therapy, as well as befriending, although no evidence was found in support of befriending as being superior to other interventions. Within this approach, befriending stands as an intervention of interest, still, as it is one of the most easily available approach. It does not involve extensive training nor does it come with associated risks, and it is hypothesized to be an effective intervention for individuals with schizophrenia as it serves as a resource that these patients can utilize in order to better integrate in the community.

Befriending is a one-on-one companionship that is regularly provided by a volunteer who aims to act as an emotionally supportive liaison (3). Although concepts such as mentoring, peer support and friendship are considered related to befriending, and sometimes used interchangeably, there are important distinctions the literature highlights: mentoring aims at achieving specific goals; peer support implies that the provider has lived experience of the condition, while friendship refers to a private, mutual and voluntary exchange between two individuals (4). The befriending interventions are not responsible for targeting particular dilemmas or symptoms nor do they imply deeper, private, mutual connections; rather it is simply a social interaction between patients and volunteers, hence volunteer training is not compulsive (3). The discussed topics are generally of a neutral nature, including possible interests and hobbies of the patient; thus, the volunteer would not challenge any delusions of the patients with schizophrenia and would instead divert the conversation to a different, neutral topic (5). Due to the ease of initiating and low cost involved, this type of intervention, befriending as a type of treatment protocol has the potential to be widely used in all clinical and community settings for patients with schizophrenia.

While befriending may have been exercised between patients and healthcare workers or volunteers for quite some time already, the effects of befriending on individuals with schizophrenia and their symptoms have not received extensive academic attention. In the systematic review on studies occurring before 2009 (6), only one literature source was identified that was at all related to befriending in treating early psychosis, although the onset of schizophrenia occurs at an early, socially vulnerable stage of life. Additionally, befriending was only used as a controlled condition, meaning no study was identified prior to 2009 examining the effect of befriending as an intervention on early psychosis.

In this scoping review, the aim is to investigate the existing literature regarding befriending either as an intervention or a controlled condition in studies on schizophrenia to gather the findings and conclusions on the effect of befriending on patients with this diagnosis and their associated symptoms. In further considering the limitations of these studies, the hope is to also delineate if or how befriending as an intervention is useful in individuals with schizophrenia, as well as to provide directions and considerations for future studies that should be conducted.

## Methods

Befriending has only recently begun to gain academic traction as it has been a less utilized, and therefore less studied, intervention style in the past. To ensure the broadest scope allowing for the identification of the greatest number of evidence-based studies, the literature search involved four databases: PubMed, Medline, APA PSYCinfo, and Embase. To be considered eligible for this scoping review, articles must have presented experimental results using befriending either as an intervention or as a controlled condition for individuals with schizophrenia.

On all four of the databases the keywords “befriending” and “schizophrenia” were used with the Boolean operator “AND.” As studies examining befriending as an intervention are fairly limited, the publication dates were set to include “all publications since 2000”. Only articles published in peer-reviewed journals with full text available are included in this scoping review.

**Figure 1:**
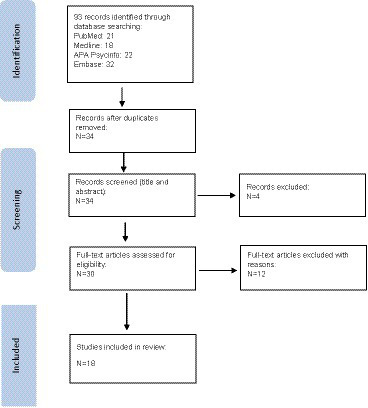
PRISMA flow diagram for befriending studies in patients with schizophrenia.

The initial PubMed database search returned 21 results, Medline database 18; APA PSYCinfo database 22 and the Embase database search returned 32 results. Following the elimination of duplicate articles, the ones examining the effect of befriending interventions on individuals other than those with schizophrenia, articles examining only interventions other than befriending on individuals with schizophrenia, and other systematic reviews themselves, a total of 18 studies meeting the criteria described above were examined in the present scoping review (see Figure 1). The studies in the current review include: (1) 11 randomized controlled trials; (2) 1 exploratory non-controlled study; (3) 1 prospective, single-blind, randomized controlled trial; (4) 1 parallel, single-blind, randomized controlled trial; (5) 1 parallel, randomized controlled trial; and (6) 1 preliminary controlled/comparative study.

## Results

The studies included in this review have all incorporated befriending as an intervention or a controlled condition, as per our search criteria, and aimed at depicting the value and feasibility of this intervention to address social and clinical deficits in individuals with schizophrenia.

Of the 18 articles selected, 7 are secondary research studies – performing statistical analysis on data from other studies, included here: (9–13) use data from (7), Allot et al. (15), and (8) use data from (18).

Ten of the articles describe randomized controlled studies [(19); 7); (1, 18, 20–25)] and one an exploratory non-controlled study (26).

The population in most of the studies refers to individuals with schizophrenia except for Jackson et al. (18) that refers to individuals with first episode of psychosis and Botero-Rodriguez et al. (26) that includes individuals with bipolar disorders along with individuals with schizophrenia. A summary of main protocol, outcome measures and key findings in each article included can be seen in Table 1.

**Table 1:** Study and participant characteristics, protocol, outcome measures, and key findings in the selected studies.

Publication Author, Year	Study design participants	Protocol	Outcome measurements	Key findings
(19)	Randomized controlled trial *N* = 19 participants with schizophrenia CBT = 13 BF = 6	6 sessions over 2 months Avg. 20–40 min Outcome assessment points: (1) baseline, (2) 1-month and (3) 2-months	Outcome measurements: (a) the time spent as a hospital in-patient during the 6 months from the commencement of individual participation, (b) CPRS and MADRS	1. The BF group showed improvement in symptoms but not significant; 2. The CBT group showed more marked improvements with significant differences in the reduction in global CPRS scores compared to the BF group; 3. Non-significant trend towards shorter stay in the hospital in the CBT group.
(7)	Randomized controlled trial *N* = 90 participants with schizophrenia resistant to medication CBT = 46 BF = 44	19 treatment sessions over 9 months 45 min/session Outcome assessment points: 1.Baseline 2.End of therapy 3.9 months follow-up	CPRS, MADRS, SANS	1. CBT and BF resulted in significant symptom improvements at the end of the treatment period but there were no significant differences between the two interventions 2. At 9-month follow-up CBT resulted in significantly greater improvements for all outcome measures; 3. Both treatments led to substantial percentages of patients improving, with CBT superior to BF in reducing CPRS scores
Milne et al. (11)	Secondary research study Archival data: 20 adult female participants from (14) study, and 20 participants from (7) study	Correlation conducted between the sample of (7) BF data and the archival social support data; Cross-sectional comparison between the two conditions in the (7) study to determine divergence	SOS; Sampling analysis of the therapists’ speech content for each intervention	1. BF intervention was similar to “social support” (associated with companionship and emotional support) and not a diluted form of CBT; 2. BF was an effective short-term intervention.
(11)	Secondary research study Comparative study between: 1. London Newcastle (LN) study (7) *N* = 90 CBT = 47 BF = 43 2. Insight into Schizhophrenia (IS) study (16) CBT = 257 Treatment as usual = 165	Aim of the analysis: to assess improvement of anxiety with CBT Patients in both studies received a CBT-based intervention in the treatment group; The control group in the LN study received BF; in the IS study – treatment as usual	CPRS, Brief Scale for Anxiety, Insight Scale, Health of the Nation Outcome Scale	1. In LN study the differences in anxiety scores between CBT and BF groups were statistically significant only at follow-up; 2. In the IS study statistically significant differences were obtained between the treatment and control group; 3. CBT was deemed to have an antipsychotic as well as anxiolytic effect, which persisted once therapy has stopped.
(9)	Secondary research study Samples from Senksy et al. (2000) Randomized controlled trial *N* = 90; CBT = 46, BF = 44	Statistical analysis based on the original protocol from (7): 19 intervention sessions over 9 months	Suicidality measured using item 7 on CPRS – “suicidal thoughts”	1. Decrease in suicidality from baseline to follow-up for both the BF and CBT interventions; 2. Significant within-group reduction only in the CBT group; 3. No significant differences between the intervention groups at baseline, but significant differences at post-treatment and follow-up.
(12)	Secondary research study Samples from Snenky et al. (2000)-Randomized controlled trial Patients with schizophrenia CBT = 46 BF = 44	Statistical analysis based on the original protocol from (7): 19 intervention sessions over 9 months	Delusions and hallucinations assessed with CPRS and entered in two linear models as predictors of treatment response	1. Neither symptom – delusions or hallucinations predicts treatment response by the end of intervention; 2. Both symptoms strongly linked to outcome at short-term follow-up
(13)	Five-year follow-up on (7), (randomized controlled trial *N* = 59; CBT =31, BF = 28)	Measurements used in the original study were repeated at 5 year follow-up	Main outcome measurements: CPRS, MADRS and SANS	1. Both groups showed an improvement in the PANSS positive subscale over time; 2. Both groups showed no improvement in the PANSS negative subscale; 3. CBT group showed a significant difference over time for the total score of the PANSS and QoL, but the BF group showed no difference in either scales; 4. CBT is superior to BF in reducing psychotic symptoms.
(20)	Preliminary controlled study/comparative study *N* = 21 patients with schizophrenia, refractory to clozapine CBT = 12 BF = 9	20 sessions over 21-weeks The first 15 sessions were performed on a weekly basis and the last 5 sessions were performed every other week. Mean: 45 min Outcome assessment points: (1) baseline, (2) week 7, (3) week 14, and (4) week 21	Outcome measurements: BPRS-Anchored version, PANSS, CGI, and QoL	1. Significant reduction in the mean scores in the CBT group as compared with BF group over time in BPRS, PANSS General Psychopathology subscale and CGI throughout the study period; 2. Both groups showed a decrease in the PANSS positive subscale over time; 3. The PANSS negative subscale showed no statistical significant differences either between or within-subjects; 4. Patients who received BF did not continue to improve at 9-month follow-up whereas CBT group did.
(18)	Randomized controlled trial *N* = 62 participants with first episode of psychosis; ACE = 31, BF =31	Max. 20 sessions over 14 weeks Approx. 45 min/session Outcome assessments points: (1) pre-treatment, (2) mid-treatment, (3) end-of-treatment and (4) 1-year follow-up	Outcomes measurements: (a) the psychotic subscale of BPRS, (b) SANS, (c) SOFAS	1. Improved functioning to a greater degree for ACE than for BF group at mid-term; 2. No differences in positive and negative symptoms between the groups by the end of the intervention; 3. ACE did not produce differential outcomes over BF at 1 year follow-up on any outcome measure.
(15)	Secondary research study Samples from Jackson et al. (18) (Randomized controlled trial *N* = 62; CBT = 31, BF =31)	Series of regressions conducted for each group to examine patient-related variables predicting positive and negative symptoms and social functioning at 1 year-follow-up	Outcome variables: (1) the level of positive symptoms (BPRS Psychotic subscale), (2) the level of negative symptoms (SANS Total score), and 3) the level of social and occupational functioning (SOFAS score) at 1-year-follow-up	The predictors of symptom and functional outcome differed for the CBT and BF groups; 1. Poorer functioning at baseline was associated with poorer symptom and functional outcome at 1-year follow-up in the CBT group; 2. Poorer premorbid adjustment predicted poorer symptom and functional outcome in the BF group.
(8)	Secondary research study Samples from Jackson et al. (18) (Randomized controlled trial *N* = 62; CBT = 31, BF =31)	Examined weather BF controlled for non –specific factors when compared to CBT: time in therapy, expectancy created by therapy and acceptability of therapy	Satisfaction Questionnaire (developed for this study, not psychometrically validated)	1. Time in therapy: both therapies received similar number of sessions; however BF participants received shorter therapy sessions and were less satisfied with the length of therapy; 2. Expectancy: high levels of expectancy in both groups – participants convinced of the utility of both interventions at the end of treatment; 3. Acceptability: both interventions were well accepted by participants.
(24)	Prospective, single-blind, randomized controlled trial Total participant *N* = 44 individuals experiencing command hallucinations TORCH = 12, BF = 14 Waitlist participants: TORCH = 9, BF = 8	15 weekly sessions 2 follow-up sessions Approx. 50 min Outcome assessment points: 1.pre-intervention; 2. end of therapy; 3.6 months follow-up	Primary outcome measures: (a) ratings of degree of compliance by assessors, (b) Self-rating of confidence to resist obeying harmful commands and 3) confidence in coping with general commands Secondary outcome measures: (a) PANSS, GAF, (b) SHER c) PSYRATS (Auditory Hallucinations), (d) Q-LES-Q, (d) subjective feedback, (e) VAAS, (f) BAVQ-R, (g) RSQ	1. Compliance with harmful command hallucinations proved not to be a viable outcome measure; 2. No differences were found between or within the groups on confidence to resist harmful commands at endpoint; 3. No differences were found between the groups on confidence in coping with command hallucinations at endpoint; both groups improved in confidence at endpoint compared to baseline; 4. TORCH group showed significant improvements across the PANSS variables and Modified GAF; 5. BF group showed significant improvement on the distress variables; 6. Both groups improved in quality of life variables – with more robust improvement in TORCH.
(21)	Randomized controlled trial *N* = 54 participants with schizophrenia spectrum disorders; BF = 29, Art Therapy = 25	Art therapy group: once-a-week sessions for 6-months 90-min /session Befriending group (control): twice-a-week sessions for 6-months 45-min/session Outcome assessment points: (1) baseline and (2) after six months	Outcome measurements: (a) CGI-Severity Scale, the 18-item Italian version of the BPRS, (b) CISS, (c) RES, GAF, (d) PSP	1. A significant improvement was observed for both interventions in psychosocial functioning, self-esteem and thought disturbance; 2. BF was found superior to Art therapy in improving psychosocial functioning; 3. BF group showed greater improvement in emotion-oriented coping strategies.
(1)	Randomized controlled trial *N* = 124 patients with schizophrenia; BF/Intervention = 63 Information only/Active control = 61	Weekly for one year Median no. of meetings: 14 (range: 1–42) Mean: 90 min Outcomes assessment points: (1) at baseline, (2) at the end of the 12-month program (3) 6-month follow-u	Primary outcome measurement: time spent in activities – using an adapted version of the TUS applied to the past 4 days.	1. Time spent in activities increased in both groups but with no differential benefit for befriending; 2. BF group had significantly more social contacts after one year – maintained 6 months after the end of the program.
(22)	Randomized controlled trial *N* = 120 participants with schizophrenia spectrum disorders; SCIT = 61, BF =59	Weekly 2 h sessions over 12 weeks SCIT participants attended an average of 6.3 sessions. BF participants attended an average of 6.6 sessions. Outcome assessment points: (1) baseline, (2) post-intervention and (3) 3-month follow-up	Primary outcome measurements: BLERT Secondary outcome measurements: (a) SFS, SSPA, HT, IPSAQ	No clinically significant differences between group outcomes on any measure of social cognition or social functioning; The improvement observed in both groups did not reach clinical significance.
(23)	Parallel, single-blind, randomized controlled trial *N* = 130 participants with persistent persecutory delusions; Feeling safe program (cognitive therapy) = 64, BF =66	Approx. 20 sessions over 6-months Outcome assessment points: (1) baseline, (2) 6 months (end of treatment), and (3) 12 months	The primary outcome measurement: PSYRATS Secondary outcomes measurements: Green et al. Paranoid Thoughts Scale, Columbia-Suicide Severity Rating Scale, BDI, DAR-5 scale, SPEQ, Warwick-Edinburgh Mental Wellbeing Scale, EQ-5D-5L, LTCQ	1. The cognitive therapy led to a significant reduction in persistent persecutory delusions compared to BF; 2. At the end of the intervention there were significant improvement in the cognitive therapy group in 7 secondary outcomes: overall paranoia, anger, ideas of reference, psychological wellbeing, patient satisfaction, time use and quality of life; 3. BF showed no significant improvement above the cognitive therapy in any outcome.
(25)	Parallel, randomized controlled trial *N* = 65 patients with schizophrenia; intervention =33, control/treatment as usual = 32 Only 55 completed follow-up assessments	Average of 5 meetings over 6-months Outcome assessment points: (1) baseline, (2) 6-months and (3) 12-months	Primary outcome measurement: MANSA Secondary outcome measurements: (a) BPRS and (b) SIX	1. Intervention had a significant effect on quality of life at 6-months and at 12-months; 2. Intervention had a significant effect on psychiatric symptoms at 6-months and 12-months.
Botero-Rodriguez et al. (26)	Exploratory non-controlled study *N* = 23 patients with schizophrenia or bipolar disorder Participants were matched into groups of 3 with 2 volunteers	12 sessions of 2 h every 15 days over 6 months Outcome assessment points: (1) baseline and (2) end of intervention (after 6 months) (3) follow-up after 12 months	Outcome measurements: (a) MANSA, (b) SIX, (c) BPRS, (d) ISMI	Large and sustained improvements in quality of life and reduction of psychiatric symptoms were observed

ACE, Active Cognitive Therapy for Early Psychosis; BF, Befriending; CBT, Cognitive Behavioral Therapy; QoL, Quality of Life; SOS, Support Observation Scale; TUS, Time Use Survey; PANSS, Positive and Negative Syndrome Scale; BDI, Beck Depression Inventory; MANSA, Manchester Short Assessment of Quality of Life; SIX, Objective Social Outcomes Index; BPRS, Brief Psychiatric Rating Scale; SANS, The Scale for the Assessment of Negative Symptoms; SOFAS, The Social and occupational functioning Assessment Scale; CPRS, The Comprehensive Psychopathological Rating Scale; SCS, Schizophrenia Change Score; CGI, Clinical Global Impression; MADRS, the Montgomery-Asberg Depression Rating Scale, GAF, the Modified Global Assessment of Functioning scale, DSM-IV version; PSYRATS, Psychotic Symptoms Rating Scale; CSQ, Client Satisfaction Questionnaire; VAAS, Voices Acceptance and Action Scale; RSQ, Recovery Style Questionnaire; BLERT, Bell Lysaker Emotion Recognition Task; SFS, Social Functioning Scale; CISS, the Coping Inventory for Stressful Situations; RES, the Rosenberg Self-Esteem Scale; PSP, the Personal and Social Performance scale; ISMI, The 29-item Internalized Stigma of Mental Illness scale; BTIM - The Befriending Treatment Integrity Measure; PDI, Peters Delusions Inventory; AAQ, the 16-item Acceptance and Action Questionnaire; TORCH, Treatment of Resistant Command Hallucinations.

## Discussions

To adequately compare and contrast results, the aforementioned studies have been divided into two categories: befriending as an intervention and befriending as a controlled condition. It appeared important to indicate the differences between the two set-ups as they involve different modalities of delivery. Befriending as an intervention itself puts greater emphasis on adjusting to client needs, including the time and place to undertake the intervention, which varies depending on the needs and interest of the client. On the other hand, befriending as a controlled condition was conducted at a fixed time and place to minimize the differences in controlled variables. Evidently, the difference in these set-ups contribute to the generation of different results.

A series of ***limitations*** of the studies included in this review challenge a straightforward understanding of the value of befriending in the mix of care strategies for individuals with schizophrenia. The small sample size not allowing for results to reach significance level, followed by the wide variety of outcome measurements and the differences in the format of delivery of the befriending interventions may all contribute to the inconsistent findings. In the study by Sikira et al. (25) for example, patients were paired with one volunteer and meetings were conducted in a one-on-one format, whereas in the study by Botero-Rodriguez et al. (26), patients were assigned to a group, with an average of 3.8 attendees in each meeting, based on similarity in interests; in the rest of the studies, although befriending happened in a one-on-one format, it was delivered by highly skilled therapists. These differences in the delivery of befriending raises the question of which is the best format for delivering the intervention as well as whether or not the delivery influences the effect of befriending on specific symptoms or functioning in patients with schizophrenia.

Studies that included *befriending as an intervention* in their design (1, 21, 25, 26) had consistently found improvements in some of the clinical or social domains assessed, with notable significant differences: (21) found a significant improvement in psychosocial functioning, self-esteem and thought disturbance; participants in the befriending group in Priebe et al. (1) study had significantly more social contacts than those in the group offered information only; Sikira et al. (25) found that the quality of life of participants in the befriending intervention significantly improved both at the end point and 12 months follow-up. Although different outcome measures were involved in these studies, their results come in alignment with the general view in the literature that befriending improves psychological and social functioning by reducing loneliness, allowing individuals to re-gain confidence and connect or re-connect with social sources of support. Of note, also is the fact that only 2 of the studies included in this review used actual volunteers for befriending: Botero-Rodriguez et al. (26) and Sikira et al. (25), the rest of the studies had the befriending delivered by the same therapists providing the main therapeutic intervention (CBT and CBT based approaches). Considering the impact of the clinical background has on the person providing the befriending, the level of expertise becomes an important confounding factor that was overlooked in these studies. When delivered by a health care professional, befriending may present as an intervention focusing on not providing therapeutic elements that would otherwise be provided, and the nature of the relationship is pre-determined by the professional – as opposed to following a natural course. At the same time it is not financially wise to use certified clinicians to perform underqualified work – unjustifiably increasing the cost of delivery.

The condition the befriending was compared to in these studies varied – from art therapy (21), to providing information on social activities only and treatment as usual [(1, 25), respectively], while Botero-Rodriguez et al. (26) explored changes within the same befriending group pre- to post intervention. Historically seen as an intervention aiming to support individuals with low levels of social support, befriending is becoming gradually more accepted and popular, and such, a certain level of expectation can be considered as a confounding factor not always addressed.

When compared to a cognitive type of therapeutic intervention – ***befriending as a control condition*** did not appear to fare as well, each study highlighting the superiority of the intervention addressing specific symptoms within a highly specific clinical approach. CBT was used in 4 of the studies included here (7–19), while 3 of the studies used interventions based on a related cognitive therapeutic approach [ACE in (18); TORCH in (24); Feeling Safe Program in (23)]. The common denominator in these interventions refers to the provision of specialized sessions requiring a highly skilled/trained clinician as opposed to the non-specific approach in befriending. Further confirming their findings, the secondary research studies (9–12, 15) highlight particular differences, according to the outcome measurements included. Notably, significant reduction in suicidality in the CBT group, compared to BF was found in the analysis done by Bateman et al. (9). Recent evidence, indicating an association between inflammatory markers and suicidality as well as the use of low doses of buprenorphine further widen the spectrum of avenues in safely addressing it (27, 28). The importance of exploring the most effective approaches in this regard remain of utmost importance.

Statistically significant differences in anxiety improvement in the CBT group at follow-up was found by Naeem et al. (11) concluding that CBT has an antipsychotic as well as anxiolytic effect, persisting after the therapy stopped.

Of particular interest, in the 5-year follow-up to Sensky et al. (7) study, Turkington et al. (5) found that although both groups (CBT and BF) showed an improvement in the PANSS positive subscale, only the CBT group showed a significant difference over time in the total score of the PANSS as well as quality of life, concluding that CBT is superior to befriending in reducing psychotic symptoms. As expected, these findings confirm the extensive literature highlighting the effectiveness of CBT in psychosis, especially in reducing positive symptoms (29). At the same time these findings acknowledge the benefits an intervention like befriending can provide, considering it was found to have similar acceptability compared to intervention groups and a high level of satisfaction reported by individuals in befriending groups (24). Also, the advantages of befriending as a control condition are not maximized as it loses its flexibility as an intervention due to its delivery at a fixed time and place to minimize the differences in the controlled variables. However, comparison between the studies is difficult as they adopted different scales to measure the same symptoms.

Outcome measures varied greatly – from clinical ones – focusing on symptom improvement, like BPRS, CPRS, PANSS, PSYRATS, SANS, and CGI, to more diverse ones focusing on quality of life, use of time, social functioning, client satisfaction and acceptance. There were also differences in the clinical populations addressed (first episode psychosis, schizophrenia, bipolar disorder), as well as duration and frequency of the intervention.

Although highly recommended by clinical practice guidelines, psychotherapeutic interventions such as CBT for psychosis require lengthy therapist training and prove difficult to be delivered on a large scale within mental health services. Continuing efforts are needed to explore supportive interventions that allow for social engagement and acceptance for this stigmatized population segment, in the context of a comprehensive socio-psychopharmacological approach.

## Conclusion

Even though a limited number of researchers focused on the effects of befriending as an intervention in schizophrenia, all 4 studies included had consistently found improvements in some of the clinical or social domains. Although different outcome measures were involved, their results confirm that befriending improves psychosocial and social functioning.

One of the main challenges of the current review was the use of different protocols and outcome measures in the selected studies on befriending as an intervention. A notable limitation we found was the apparent misuse of resources in delivering the befriending, raising the question of appropriateness in delineating what befriending is, as well as its cost-effectiveness.

When delivered by a healthcare professional, befriending may present as an intervention focusing on not providing therapeutic elements that would otherwise be provided, and the nature of the relationship becomes pre-determined by the professional – as opposed to following a natural course. At the same time, the use of certified clinicians to perform underqualified work unjustifiably increases the cost of delivery.

As expected, in studies where befriending was a control condition, results were superior for the specialized interventions condition (CBT and cognitive-based interventions) but they do not detract from the importance of befriending as evidenced by a generally positive acceptance and improvements noted during the intervention.

Although the clinical effects rarely reached significance or withstood the test of time by comparison with established psychotherapeutic interventions, befriending was identified as providing an opportunity for increased social interactions and the development of healthy social relationships, suggesting that it may be considered a complementary or supplementary intervention for patients with schizophrenia, especially when CBT is not readily available.

### Further directions

Further directions pertain to: *larger sample size studies* targeting befriending in schizophrenia with well-defined protocols, longer follow-ups (to determine the timing and frequency of maintenance sessions), using the most relevant clinical outcome measures (PANSS, QoL, third party/collateral information scales). Based on results from follow-ups we could design possible *intervention protocols* that include well-timed maintenance sessions. A possible model of maintenance session could be based on the protocol used in 26 study and include a progression of delivery of the intervention from one-on-one to a small group form. These protocols would be easy to implement in all clinical and community study, as well as cost effective, provided that the intervention is not performed by highly trained clinicians. A next step in this regard may involve the development of a minimal yet necessary *training curriculum* for volunteers. This would ensure volunteers are not to interfere with or challenge problematic symptoms, while at the same time allow them to interact with patients in a non-judgmental, accepting and hope-promoting manner.

An avenue not explored in the literature is the use of befriending as a *pre-intervention tool*, preparing patients for accepted interventions like CBT. The interactions with a volunteer in the time leading to the clinical intervention, would open the door to the therapeutic engagement essential for therapeutic change. In this context, adequate resources would be used for appropriate outcomes and ensure a more comprehensive and effective approach.

## Author contributions

All authors listed have made a substantial, direct, and intellectual contribution to the work and approved it for publication.

## Funding

This work was supported by Providence Care Innovation Grant 2019: “Innovative pathways to impactful treatment of chronic schizophrenia: disrupting the status-quo moving toward biological-driven, combined pharmacological, and non-pharmacological therapeutic approaches to define markers of therapeutic improvement in cognitive behavioral therapy for psychosis promoted recovery”.

## Conflict of interest

The authors declare that the research was conducted in the absence of any commercial or financial relationships that could be construed as a potential conflict of interest.

## Publisher’s note

All claims expressed in this article are solely those of the authors and do not necessarily represent those of their affiliated organizations, or those of the publisher, the editors and the reviewers. Any product that may be evaluated in this article, or claim that may be made by its manufacturer, is not guaranteed or endorsed by the publisher.
